# New Source of 3D Chitin Scaffolds: The Red Sea Demosponge *Pseudoceratina arabica* (Pseudoceratinidae, Verongiida)

**DOI:** 10.3390/md17020092

**Published:** 2019-02-01

**Authors:** Lamiaa A. Shaala, Hani Z. Asfour, Diaa T. A. Youssef, Sonia Żółtowska-Aksamitowska, Marcin Wysokowski, Mikhail Tsurkan, Roberta Galli, Heike Meissner, Iaroslav Petrenko, Konstantin Tabachnick, Viatcheslav N. Ivanenko, Nicole Bechmann, Lyubov V. Muzychka, Oleg B. Smolii, Rajko Martinović, Yvonne Joseph, Teofil Jesionowski, Hermann Ehrlich

**Affiliations:** 1 Natural Products Unit, King Fahd Medical Research Centre, King Abdulaziz University, Jeddah 21589, Saudi Arabia; 2 Suez Canal University Hospital, Suez Canal University, Ismailia 41522, Egypt; 3 Department of Medical Parasitology, Faculty of Medicine, Princess Al-Jawhara Center of Excellence in Research of Hereditary Disorders, King Abdulaziz University, Jeddah 21589, Saudi Arabia; hasfour@kau.edu.sa; 4 Department of Natural Products, Faculty of Pharmacy, King Abdulaziz University, Jeddah 21589, Saudi Arabia; dyoussef@kau.edu.sa; 5 Department of Pharmacognosy, Faculty of Pharmacy, Suez Canal University, Ismailia 41522, Egypt; 6 Institute of Chemical Technology and Engineering, Faculty of Chemical Technology, Poznan University of Technology, Poznan 60965, Poland; soniazolaks@gmail.com (S.Ż.-A.); marcin.wysokowski@put.poznan.pl (M.W.); teofil.jesionowski@put.poznan.pl (T.J.); 7 Institute of Electronics and Sensor Materials, Technische Universität Bergakademie-Freiberg, Freiberg 09599, Germany; iaroslavpetrenko@gmail.com (I.P.); yvonne.joseph@esm.tu-freiberg.de (Y.J.); 8 Leibniz Institute of Polymer Research Dresden, Dresden 01069, Germany; tsurkanmv@gmail.com; 9 Clinical Sensoring and Monitoring, Department of Anesthesiology and Intensive Care Medicine, Faculty of Medicine, Technische Universität Dresden, Dresden 01307, Germany; roberta.galli@tu-dresden.de; 10 Department of Prosthetic Dentistry, Faculty of Medicine, Technische Universität Dresden, Dresden 01307, Germany; heike.meissner@uniklinikum-dresden.de; 11 P.P. Shirshov Institute of Oceanology, Russian Academy of Sciences, Moscow 117997, Russia; tabachnick@mail.ru; 12 Department of Invertebrate Zoology, Biological Faculty, Lomonosov Moscow State University, Moscow 119992, Russia; ivanenko.slava@gmail.com; 13 Institute of Clinical Chemistry and Laboratory Medicine, University Hospital Carl Gustav Carus at the Technische Universität Dresden, Dresden 01307, Germany; nicole.bechmann@uniklinikum-dresden.de; 14 V.P. Kukhar Institute of Bioorganic Chemistry and Petrochemistry, National Academy of Science of Ukraine, Kiev 02094, Ukraine; lmuzychka@rambler.ru (L.V.M.); smolii@bpci.kiev.ua (O.B.S.); 15 Institute of Marine Biology, University of Montenegro, Kotor 85330, Montenegro; rajko.mar@ucg.ac.me

**Keywords:** chitin, scaffolds, biological materials, demosponges, *Pseudoceratina arabica*

## Abstract

The bioactive bromotyrosine-derived alkaloids and unique morphologically-defined fibrous skeleton of chitin origin have been found recently in marine demosponges of the order Verongiida. The sophisticated three-dimensional (3D) structure of skeletal chitinous scaffolds supported their use in biomedicine, tissue engineering as well as in diverse modern technologies. The goal of this study was the screening of new species of the order Verongiida to find another renewable source of naturally prefabricated 3D chitinous scaffolds. Special attention was paid to demosponge species, which could be farmed on large scale using marine aquaculture methods. In this study, the demosponge *Pseudoceratina arabica* collected in the coastal waters of the Egyptian Red Sea was examined as a potential source of chitin for the first time. Various bioanalytical tools including scanning electron microscopy (SEM), fluorescence microscopy, FTIR analysis, Calcofluor white staining, electrospray ionization mass spectrometry (ESI-MS), as well as a chitinase digestion assay were successfully used to confirm the discovery of α-chitin within the skeleton of *P. arabica*. The current finding should make an important contribution to the field of application of this verongiid sponge as a novel renewable source of biologically-active metabolites and chitin, which are important for development of the blue biotechnology especially in marine oriented biomedicine.

## 1. Introduction

Structural aminopolysaccharide chitin is one of the oldest biopolymers due to its presence in fungi which appeared on our planet around 2.4 billion years ago [1]. In 1811, Henri Braconnot discovered chitin in the form of an alkali-resistant fraction during his studies on higher fungi and, consequently, termed it as *fungine* (for review see [2]). The currently used term *chitin*, however, has been proposed in 1823 by Auguste Odier who used beetle cuticles to isolate similar biomaterial during alkali treatment with hot KOH solutions [3]. Chitin has been found in skeletal structures of diverse unicellular organisms (yeasts, protists) and invertebrate organisms (corals, annelids, molluscs, arthropods) with exception of crustose coralline algae; cell walls of diatoms and skeletons of sponges (see for review [4,5]). The existence of chitin within the marine demosponges and glass sponges’ skeletons was reported for the first time only in 2007 [6,7]. The first report on chitin identification in siliceous cell walls (frustules) of diatoms was carried out in 2009 [8]. Intriguingly, the presence of chitin in crustose coralline algae has been described in 2014 [9]. Nowadays, chitin has been reported in 17 species of marine [10] and in two species of freshwater sponges [11,12]. One of the special characteristics of poriferan chitin is the 3D fibrous nature, which has been recognized as a naturally prefabricated tubular scaffold that follows the morphology especially of keratosan demosponges [13,14,15]. These unique 3D architectures of such scaffolds are typical for representatives of the Verongiida order (subclass Verongimorpha, class Demospongiae) and open perspectives for their applications in waste treatment [16], tissue engineering [14,17,18], electrochemistry [19] as well as extreme biomimetics [2,20,21,22,23]. Due to the fact that manufacturing of fungi, as well as crustaceans chitin into 3D sponge-like scaffolds, is difficult and expensive, the extensive research of species-specific morphology and structure of the chitin-scaffolds of sponge origin as “ready to use” materials still remain important for practical applications.

Representatives of Verongiida demosponges contain aplystane-type and bromotyrosine-derived secondary metabolites, which is a unique feature within Demospongiae. This is a very distinct chemotaxonomic marker for all members of the order Verongiida [24,25,26]. It has been proved that bromotyrosine-derived alkaloids possess antimicrobial, antifungal, cytotoxic, and antimalarial activity (for review see [27,28,29,30,31,32,33,34]). Interestingly, only nudibranchs represent the natural predator of the verongiid sponges [35]. As reported previously [36], some of bromotyrosines also showed anti-chitinase activity. Consequently, it was suggested that bromotyrosine related compounds localized within chitinous skeletons of verongiid sponges can inhibit the chitinases of bacterial and fungal origins and in this way protect the integrity of sponge skeleton [13].

**Figure 1:**
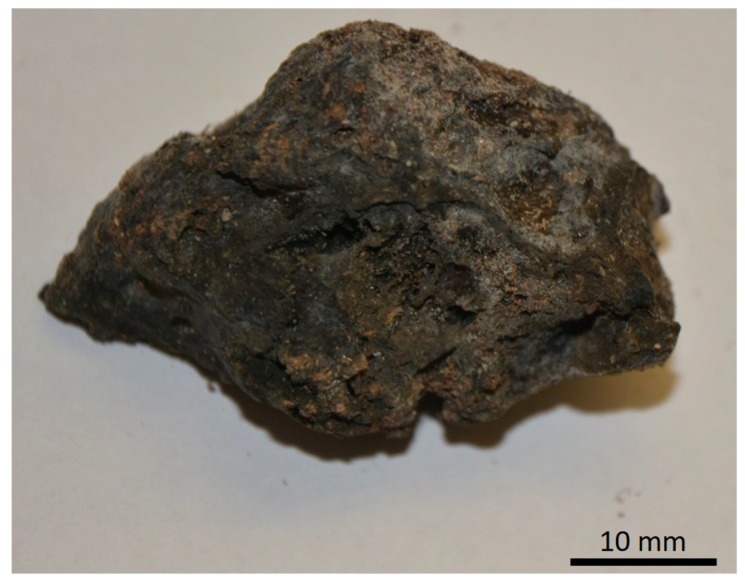
The fragment of the dried specimens of *P. arabica* demosponge used in this study.

So far, only two representative species of the Verongiida order exist in the Red Sea, namely *Pseudoceratina arabica* and *Suberea mollis*. Both sponges have been extensively investigated by our group to identify their bioactive compounds. Recently, several bromotyrosine alkaloids and halogenated compounds with different biological activities have been reported from these two sponges [27,28,29,30,31,37,38]. Due to the ability of diverse chitin-producing sponges to grow under marine ranching conditions (see for overview [39]), poriferan chitin constitutes a renewable source of such unique naturally occurring scaffolds. This encouraged studies on monitoring of novel demosponge species with chitinous skeletons. Therefore, this study focused on the bromotyrosines producing Red Sea demosponge *Pseudoceratina arabica* (Figure 1) where the presence of chitin has never been reported before.

## 2. Results

**Figure 2:**
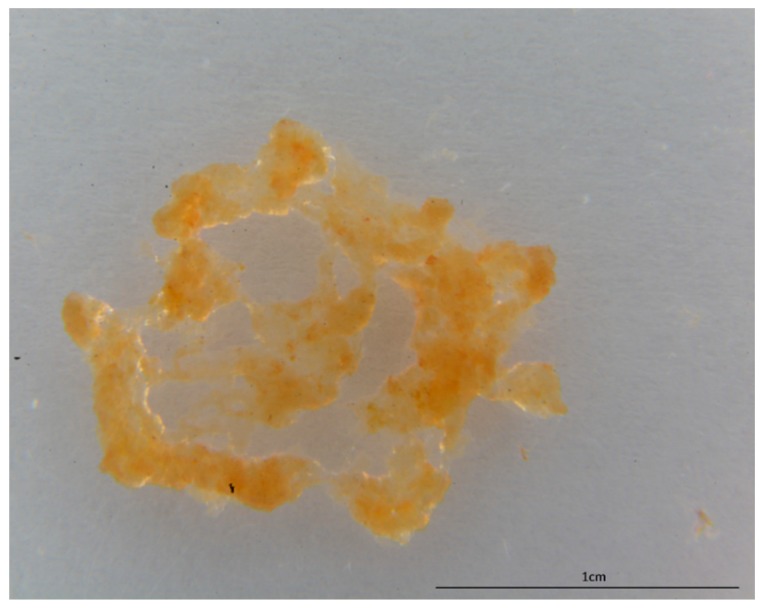
Completely demineralized and pigment-free scaffolds isolated from the sponge *P. arabica*.

**Figure 3:**
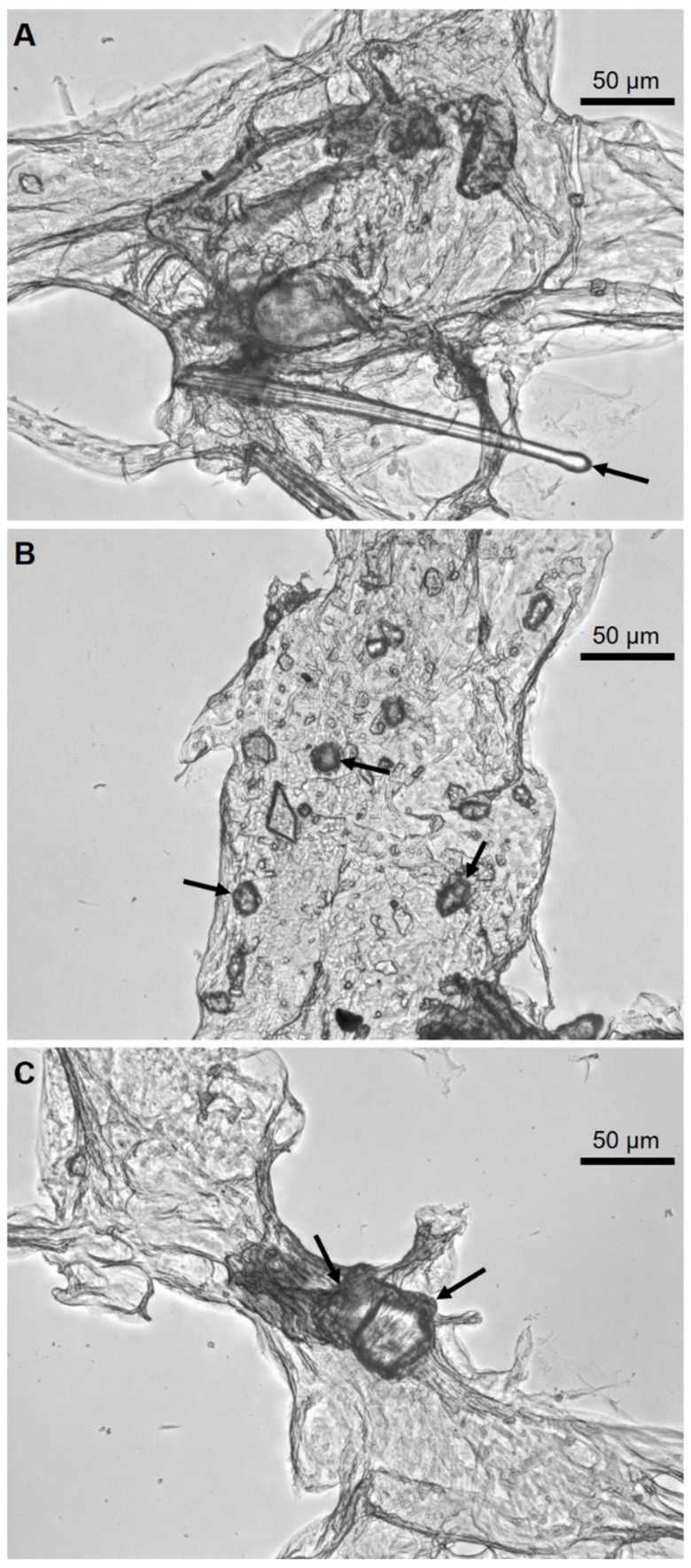
Alkali-treated fibers of *P. arabica* under the optical microscope showing foreign spicules (**A**) and microparticles of sand (**B**, **C**) (arrows).

Figure 2 clearly shows that the alkali treatment resulted in depigmented, protein-free, fibrous scaffolds with residual siliceous spicules and foreign, sandy microparticles within the fibers (Figure 3). Observations of these contaminants into the NaOH-treated fragments of *P. arabica* support our previous suggestion about the allochronic origin of sponges from Pseudoceratinidae family [33].

**Figure 4:**
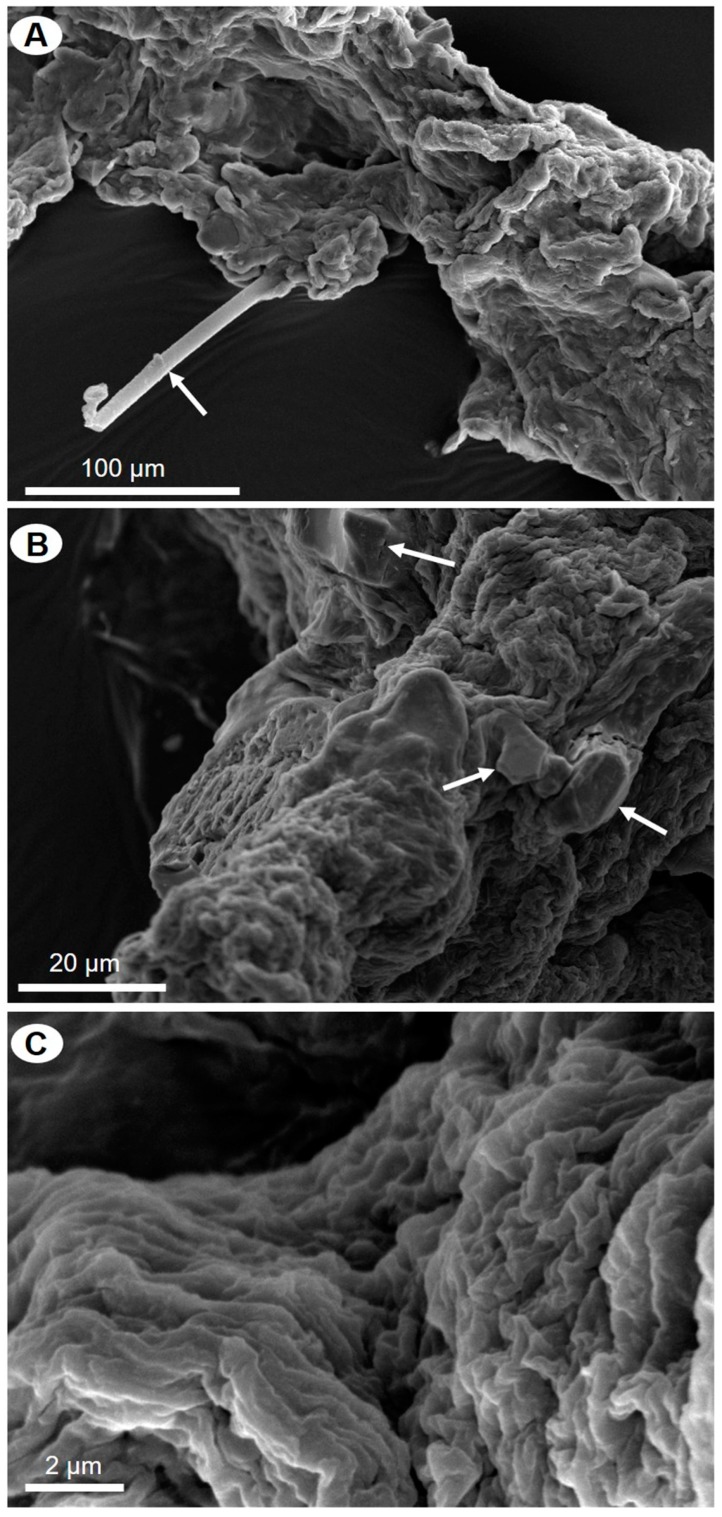
SEM images of alkali-treated skeletal fibers of *P. arabica*. Microparticles of siliceous foreign sponge spicules (**A**) and sand particles (**B**) are marked with arrows. Some parts of partially demineralized fibers remain to be free from foreign particles (**C**).

**Figure 5:**
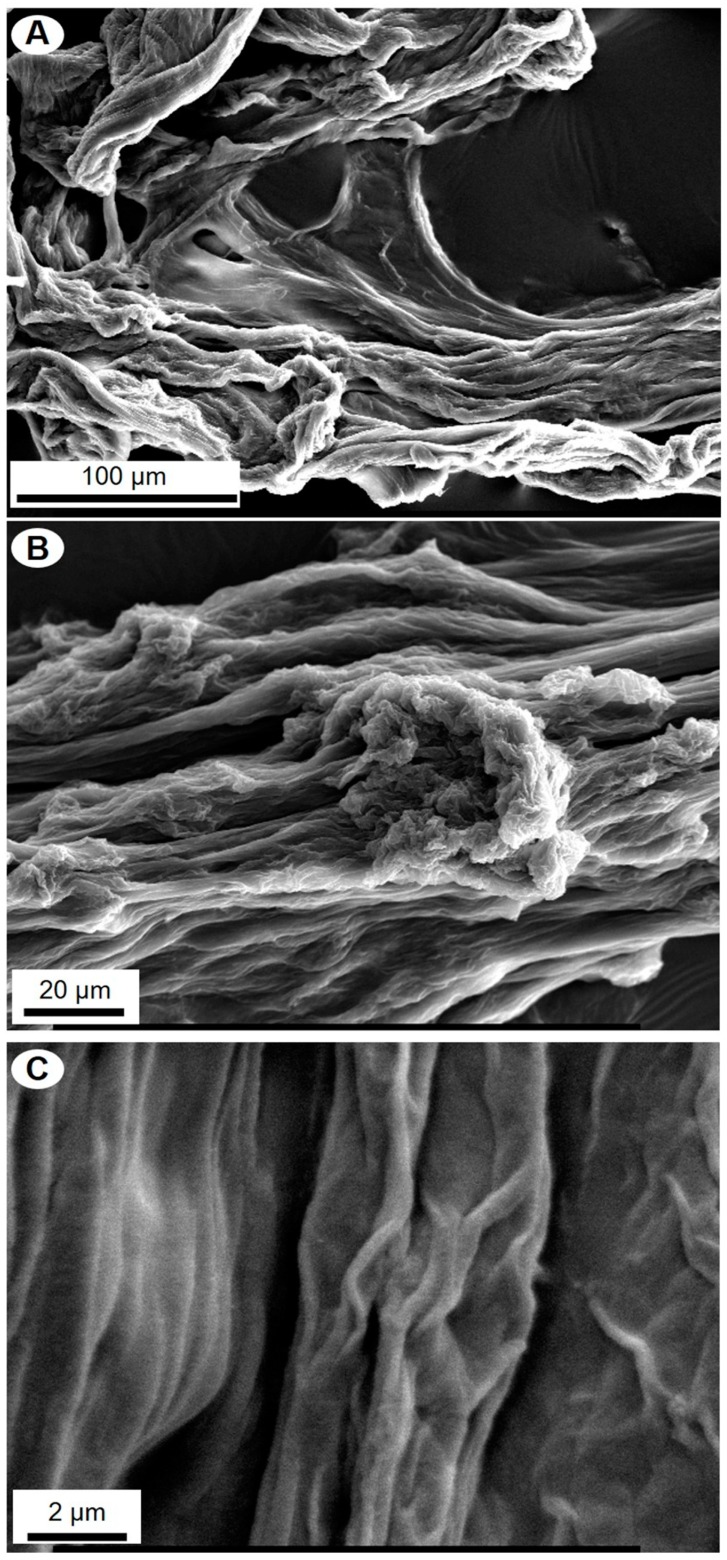
SEM images of *P. arabica* fibers after desilicification in 10% of HF under different levels of magnification (**A**–**C**).

**Figure 6:**
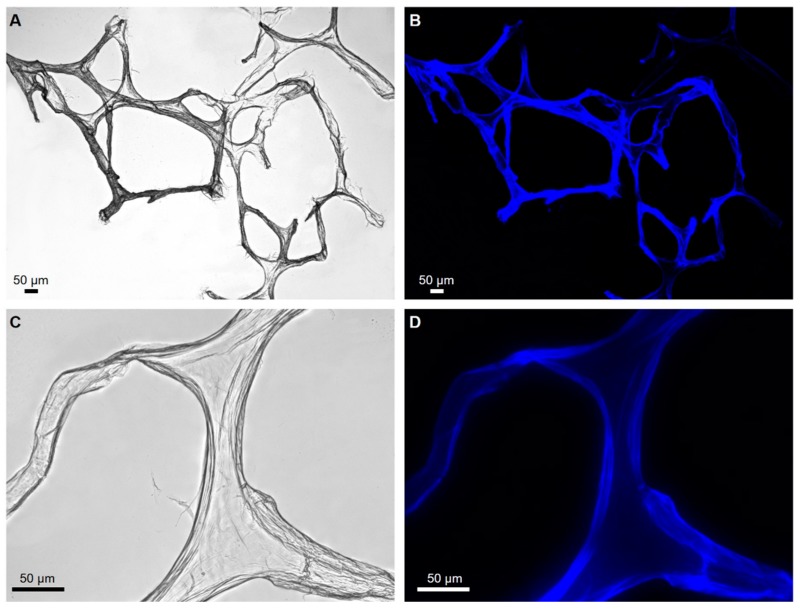
Light microscopy (**A**,**B**) and fluorescence (**C**,**D**) microscopy images of *P. arabica* fibers after desilicification in 10% HF lacking of spicules and other foreign contaminants in investigated fibers.

SEM microphotographs of the scaffolds isolated from *P. arabica* before (Figure 4) and after (Figure 5) HF-treatment show that only treatment using diluted HF water solution leads to dissolution and removal of sand microparticles as well as spicules and result in silica-free, pure, microfibers with high structural integrity, as observed before in other verongiid sponges [6,13,15,40] (Figure 5). These results were also confirmed using light as well as fluorescent microscopy (Figure 6).

Typically, Calcofluor white staining (CFW) was used as the first stage of chitin identification in completely demineralized (including HF-based treatment) sponge skeletons. This fluorescent dye is commonly used for staining β-(1→3) and β-(1→4) linked polysaccharides including chitin. Consequently, after binding to polysaccharides, CFW dye exhibits bright blue light under UV excitations [41].

**Figure 7:**
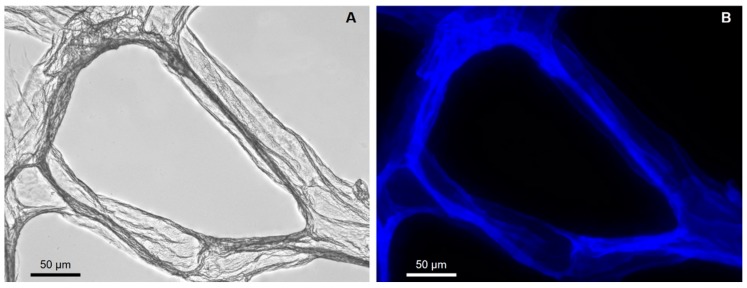
Completely purified fibers of *P. arabica* after CFW staining: (**A**) light microscopy image and (**B**) fluorescence microscopy image of the same location (light exposure time 1/4800) confirm the chitinous nature of the fibers.

Examination of the scaffolds isolated from *P. arabica* after CFW staining using fluorescent microscopy demonstrate strong fluorescence under light exposure time as short as 1/4800 s (Figure 7B). Similar conclusions were reported previously for chitin isolated from sponges of marine [6,10,15,32,33,40] as well as freshwater [12] origin and fossilized chitin-containing remains [11,41].

More precise methods were applied to study in details the presence and identification of chitin in isolated scaffolds. FTIR spectroscopy is considered as an effective technique for structural analysis of different polysaccharides including chitin. Recently, FTIR analysis was successfully used to obtain information about of type of polymorph form of chitin [42].

**Figure 8:**
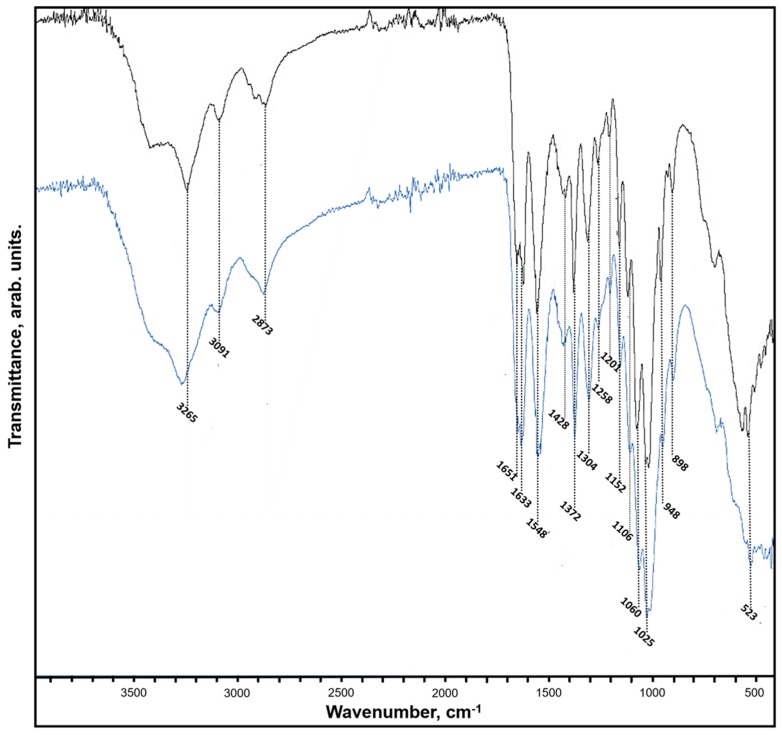
FTIR spectra of the chitin isolated from *P. arabica* compared to standard *a*-chitin.

The acquired FTIR spectra of demineralized scaffolds of *P. arabica* and standard α-chitin are presented in Figure 8. Between 1700 and 1500 cm^−1^, the different signatures characteristic for chitin polymorphs were observed. In this amidic moiety region, the investigated sample showed strong band related to the stretching vibrations of C=O group characteristic for band I of the amidic moiety. This band, registered for studied sample, possessed twin peaks at 1651 and 1633 cm^−1^, which is related with the presence of two types of carbonyl groups within the chitin chain, and it is also typical for α-chitin. The first peak derives from the specific intermolecular hydrogen bond of carbonyl group and hydroxymethyl group on the next chitin residue in the same chain. The second peak is a result of the intramolecular hydrogen bonds of carbonyl with the amide groups. Additionally, in the purified sponge chitin sample, as well as in the α-chitin standard, the characteristic intense band at *v_max_* 948 cm^−1^ which is referred to γCH_x_ bond was observed. Moreover, the α-chitin characteristic band assigned to β-glycosidic bond at 895 cm^−1^ is well visible in the studied samples. However, it should be noted that the characteristic bands for CaCO_3_ (855–876 cm^−1^) and SiO_2_ (720 cm^−1^) were not observed in the spectrum of *P. arabica*, suggesting that procedure of chitin isolation resulted in chitin of high purity. Additionally, the comprehensive analysis of acquired spectra shows that recorded bands correspond with those referred in the α-chitin reference sample.

**Figure 9:**
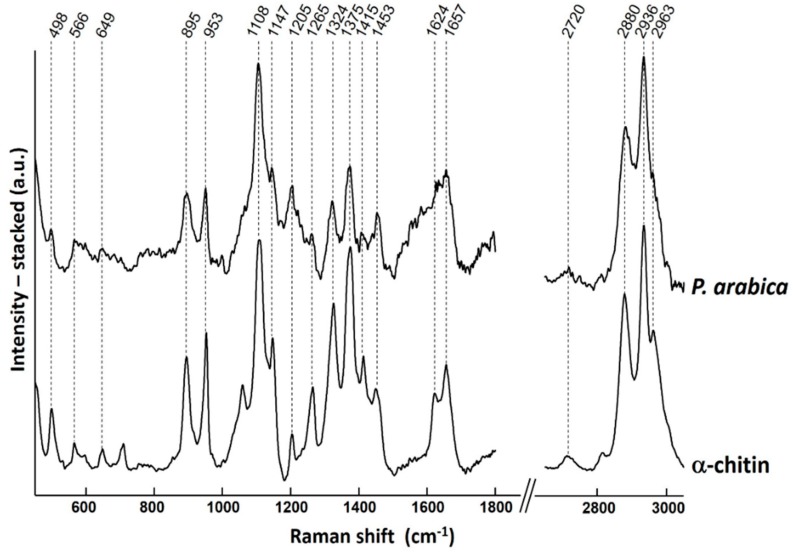
A Raman spectrum of chitin isolated from *P. arabica* compared with the spectrum of reference α-chitin. The bands of *P. arabica* are in good agreement with those of α-chitin standard within the spectral resolution of the measurements.

Figure 9 shows the Raman spectrum of chitin isolated from *P. arabica* compared with the spectrum of the α-chitin reference. Characteristic bands for α-chitin can be found in the spectrum of the isolated chitin within the spectral resolution of the measurement. The existence of two bands characteristic to amine band I at *v_max_* 1657 and 1624 cm^−1^ as well as intense band related to the β-glycosidic bond at *v_max_* 895 cm^−1^ clearly indicate that chitin isolated from *P. arabica* is of α isomorph. Moreover, the bands in the spectrum are in good agreement with previously published data [5,10,43,44].

**Figure 10:**
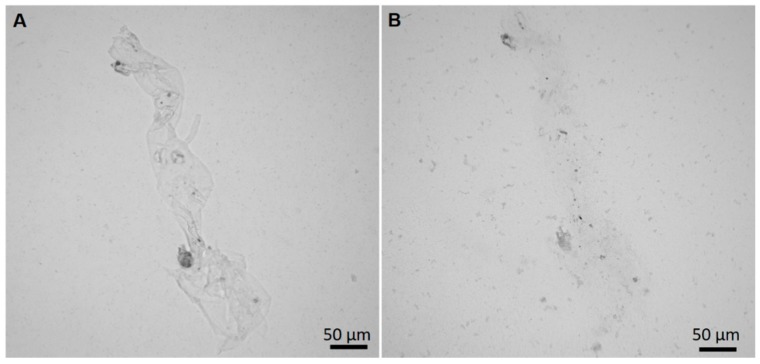
Results of chitinase digestion test on the purified skeletal fibers of *P. arabica*. Fibers before the digestion (**A**) and after 2 h of treatment with chitinase solution (**B**) are well visible.

Previously, in order to confirm the presence of chitin in diverse sponges, the chitinase digestion test has been successfully applied [6,10,15,32,33,40]. This enzyme has unique ability to decompose chitin into low-molecular oligomers such as *N*-acetyl-*D*-glucosamine (GlcNAc). Therefore, the action of chitinase leads to the loss of chitin integrity and the release of residual chitin microfibers of steadily decreasing size. The changes in the structure of treated fibers can be observed using light microscopy (Figure 10). This test is unequivocal and provides additional confirmation of the successful chitin isolation from the sponge under study here.

D-glucosamine (dGlcN) is the product of chitin’s acidic hydrolysis which can be readily identified by electrospray-ionization mass spectroscopy (ESI-MS) measurements. Thus, ESI-MS spectroscopy becomes a standard method for chitin identification which usability was shown in complex organisms [40,45,46] and even in 505-million-year-old chitin-containing fossil remnants [47].

**Figure 11:**
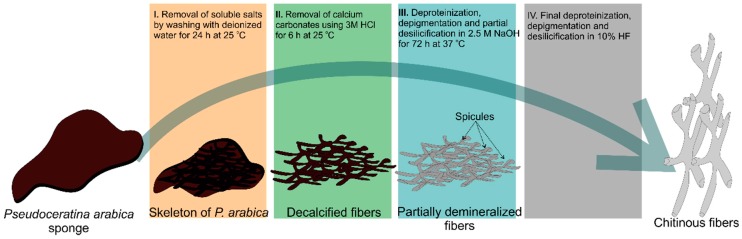
Schematic diagram showing the step-by-step procedure of chitin isolation from the skeleton of *P. arabica*.

**Figure 12:**
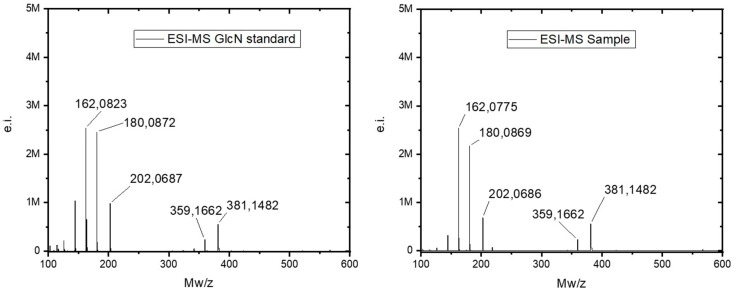
The positive ESI-MS spectra of *D*-glucosamine (dGlcN) standard (**left**) and of acid-hydrolyzed chitin (**right**) from *P. arabica*.

In the positive ESI-MS spectra, D-glucosamine (dGlcN) standard revealed several main ion peaks with *m*/*z* = 162.08, 180.09, 202.07, 359.17, and 381.15 (Figure 11). The ion peak at *m*/*z* = 180.09 and 202.07 correspond to a [M + H]^+^ and [M + Na]^+^ species with molecular weight of 179.09 which is dGlcN molecule (calculated: 179.1). The ion peak at *m*/*z* = 161.85 corresponds to a [M + H]^+^ specie with molecular weight of 160.85 that is dGlcN ion [M−H_2_O + H]^+^ without one water molecule (calculated: 161.1). There are also week ion peaks at *m*/*z* = 359.17 and 381.15 corresponding to [2M + H]^+^ and [2M + Na]^+^ species which are proton- or sodium-bound dGlcN noncovalent dimmer. The ESI-MS spectra of the *P. arabica* hydrolysate has revealed nearly identical ion peaks to those of the *D*-glucosamine standard signal composition (Figure 12). This result clearly demonstrates the presence of dGlcN in the hydrolysate and correspondingly chitin in the sample.

## 3. Discussion

Seas and oceans are a huge source of various invertebrate animals with potential to be used in biomedicine. For this reason, these organisms are frequently being tested for the presence of various useful products (unique secondary metabolites, biopolymers and biological materials), and many of them have been found in marine sponges. The order Verongiida has been recognized to be divided into four families, which differ in the structure and composition of skeletal fibers [48,49]. The largest verongiid family is Aplysinidae (52 species from three genera: *Aiolochroia*, *Aplysina*, and *Verongula*). This family is characterized by an anastomosing fiber skeleton with both pith and bark elements. The second largest verongiid family is Ianthellidae (21 species in four genera: *Anomoianthella*, *Hexadella*, *Ianthella*, and *Vansoestia*). The presence of eurypylous choanocyte chambers is a feature distinguishing this family from the others verongiids. Aplysinellidae includes 17 species in three genera (*Aplysinella*, *Porphyra*, and *Suberea*) with dendritic fiber skeleton possessing both pith and bark elements, which are typical morphological features characteristic for representatives of this family. The verongiid *P. arabica* (Keller, 1889) that has been investigated in this study belongs to the family Pseudoceratinidae, which is currently including four species representing the only genus *Pseudoceratina*. Representatives of this family are characterized by a dendritic fiber skeleton with only pith elements. Interestingly, sponges of the genus *Pseudoceratina* are assumedly the richest sources of pharmacologically active alkaloids with diverse chemical skeletons within the order Verongiida [33]. Among various secondary metabolites isolated from *Pseudoceratina* species are: moloka’iamine derivatives, phenolic halogenated compounds, psammaplysins, pseudoceratinamide A and B, ceratinines, moloka’iakitamide, aplysterol, and aplysamine [33]. To date, a variety of secondary metabolites obtained from *P. arabica* have been purified using the solvent extraction method. Surprisingly, there are no literature reports on the extraction of these metabolites using the alkaline-based solution as well as about structural stability of such biomolecules at pH above 7. Alkaline stepwise extraction procedures were recently reported as effective methods for isolation of chitin-based scaffolds with bromotyrosines from other representatives of the order Verongiida and to “squeeze the full potential” of marine sponges [39]. However, it is necessary to prove, the pharmacological and biotechnological potential of the Red Sea verongiid sponges especially because of the recently published intriguing results concerning anti-tumorigenic and anti-metastatic activity of Aeroplysinin-1 which is one of the main bromotyrosine derivatives extracted from Verongiida [34]. All Verongiida sponge samples analyzed until now were found to exhibit a chitin-based scaffold, and here it was proved that *P. arabica* is the new example of chitin-containing sponge from this order. Apart from the bioactive metabolites of *P. arabica*, which are excellently described in the literature, here it was strongly demonstrated that this marine sponge can be effectively used also as a source of naturally prefabricated 3D chitinous scaffold with open-pore structure. Unfortunately, there is still lack of information concerning the interrelationships between the secondary metabolites and chitinous skeleton of *P. arabica*, especially with respect to their localization within so called spherulous cells. However, it known that spherulous cells are rich on bromotyrosines and have been found within skeletal fibres of verongiids [50]. The questions about the role of bromotyrosines in regeneration of chitinous skeleton as well as the growth rate of this species are still open. However, these data are crucial for the future estimation of the biotechnological, biomedical and pharmaceutical potential of *P. arabica* in the region.

It is worth to mention that, the 3D macroporous biomaterials of sponge origin gain a particular interest in tissue engineering, water purification, catalysis, and electrochemistry [51]. Preliminary research done with the use of corresponding 3D chitinous-scaffolds isolated from *A. aerophoba* [17] and *Ianthella basta* [18] confirm their biocompatibility with human mesenchymal stromal cells; supporting their adhesion, viability, growth, and proliferation. Additional, useful features of chitinous scaffolds of poriferan origin are their simplicity and ease of isolation. Calculated swelling capacity for chitinous matrices isolated from *P. arabica* is equal to 255 ± 8%. There are no doubts that comparative studies on interconnected porosity and swelling ability between chitinous matrices of *P. arabica* and that from other verongiid species [17,18] should be carried out. Consequently, the discovery of chitin in other members of the genus *Pseudoceratina* would be the next stage in the evaluation of the possibility to accept these organisms as a new source of 3D chitin scaffolds with macroporosity which range between 150–350 µm for biomedical applications. We suggest that the opportunity for ex situ cultivation of *P. arabica* can be an important advantage, which enables the use of this sponge for large scale applications in diverse advanced technologies.

## 4. Materials and Methods

### 4.1. Collection of Samples

Specimens of marine sponge *Pseudoceratina arabica* (Keller, 1889) (Porifera: Demospongiae: Verongiida: Pseudoceratinidae) described initially as *Psammaplysilla arabica* Keller, 1889 were collected by hands using SCUBA from the southern part of the Egyptian Hurghada (N 27°02′46.8″ E 33°54′21.4″) in July 2017 at depths up to 25 m. The yellowish green encrusting sponge with its conulose surface measuring about 1–2 cm thick. The preserved sponge in ethanol is completely black in color with dark-discolored ethanol. The conules on the sponge surface are bluntly rounded in shape, compressible and rubbery. The individual conules measure about 2–5 mm. The sponge skeleton consists of irregular and scattered fibers composed of pith. The outline and branching were irregular with thickness measuring between 80 and 300 µm. The sponge is similar to the sample collected in Red Sea from Eritrea. The sponge voucher (10.0 × 4.0 × 1.0 cm) was kept in the Zoological Museum of the University of Amsterdam with reference no. 17951. A similar specimen of the sponge was kept at Suez Canal University with collection reference DY-61. Collected specimens were kept on ice after collection. After returning to our laboratory, the specimens were freeze-dried (Figure 1) and transferred to the bioanalytical laboratories at TU Bergakademie Freiberg (Freiberg, Germany).

### 4.2. Isolation of Chitin from P. Arabica

The isolation of chitin scaffolds from *P. arabica* (Figure 1) was carried out according to our previous reports [12,40,52]. The methodology consists of four steps (Figure 11): first, the skeleton of *P. arabica* was incubated in deionized water at room temperature for one hour to remove possible water-soluble sediment particles and salts. In the second step, the samples were treated with 3 M HCl at room temperature for 6 h in order to eliminate possible residual calcium carbonate-based debris (micro fragments of crustacean carapaces and mollusc shells) from the skeleton of *P. arabica*. Afterwards, the samples were washed several times with deionized water until achieving a pH of 6.5 followed by treatment with 2.5 M NaOH at 37 °C for 72 h to remove pigments and proteins. Due to the observation of the foreign spicules and their fragments in the samples after 72 h of alkali treatment, additional desilicification was needed. Consequently, alkali-treated samples were accurately rinsed with deionized water and stored in a plastic vessel containing appropriate amount of 10% hydrofluoric acid (HF) solution (step four). The vessel was covered in order to prevent the evaporation of HF. The desilicification process was conducted at room temperature for 12 h. The influence of alkaline and strong acidic treatments on the structure of skeleton of the studied sponge was investigated using stereo, white light and fluorescence microscopy. Finally, the isolated material was washed several times with deionized water up to a pH level of 6.5. The fibrous translucent scaffolds (Figure 2) were placed into 250 mL large GLS 80 Duran glass bottles containing deionized water and stored at 4 °C for further analyses.

### 4.3. Light and Fluorescent Microscopy Analyses and Imaging

Collected sponge samples and isolated chitinous scaffolds were observed using BZ-9000 microscope (Keyence, Osaka, Japan) in the light as well as in the fluorescent microscopy modus.

### 4.4. Scanning Electron Microscopy Analysis

The morphology and microstructure of isolated and purified chitinous scaffolds, as well as untreated samples of *P. arabica*, were studied on the basis of SEM images using a Philips ESEM XL 30 scanning electron microscope (FEI Company, Peabody, MA, USA). Before analysis, samples were covered with a carbon layer for one minute using an Edwards S150B sputter coater (BOC Edwards, Wilmington, MA, USA).

### 4.5. Calcofluor White Staining Test

Due to the fact that Calcofluor White (Fluorescent Brightener M2R, Sigma-Aldrich, Taufkirchen, Germany) exhibits enhanced fluorescence after binding to chitin [53,54], this staining method was applied to investigate the location of chitin in the completely purified fibers of *P. arabica*. The selected chitinous fibers were soaked in 0.1 M KOH-glycerine-water solution and few drops of the 0.1% CFW solution were added. This mixture was incubated for 3 h in darkness, washed several times with demineralized water, dried at room temperature and examined using BZ-9000 microscope (Keyence, Osaka, Japan).

### 4.6. FTIR and Raman Spectroscopy

FTIR spectra of chitinous scaffolds were acquired using a Nicolet 210c FTIR spectrometer. The samples were analysed using the ATR system with resolution equals 4 cm^−1^. A micro-Raman system composed by a spectrometer (RamanRxn1™, Kaiser Optical Systems Inc., Ann Arbor, MI, USA), a 785 nm excitation laser diode (Invictus 785, Kaiser Optical Systems Inc., Ann Arbor, MI, USA) and an upright microscope (DM2500 P, Leica Microsystems GmbH, Wetzlar, Germany) was used to acquire the Raman spectra from the sample surface. Each spectrum was registered in the range 150–3250 cm^−1^ with resolution of 4 cm^−1^, using a total acquisition time of 80 s. The fluorescence background was subtracted in MATLAB (MathWorks Inc., Natick, MA, USA) with a baseline procedure.

### 4.7. Chitinase Digestion Test

Yatalase® from culture supernatants of *Corynebacterium* sp. OZ-21 (Cosmo Bio, Tokyo, Japan) was used for the digestion test. Yatalase is a complex enzyme, consisting mainly of chitinase, chitobiase and β-1,3-glucanase. One unit of this enzyme released one μmol of *N*-acetylglucosamine from 0.5% chitin solution and 1 μmol of p-nitrophenol from p-nitrophenyl-*N*-acetyl-β-*D*-glucosaminide solution in 1 min at 37 °C and pH 6.0. The selected, completely demineralized chitinous scaffolds of *P. arabica* (Figure 3) were incubated in an enzyme solution containing 10 mg/mL Yatalase dissolved in phosphate buffer at pH 6.0 for 2 h. The progress of digestion was monitored under light microscopy using BZ-9000 microscope (Keyence, Osaka, Japan).

### 4.8. Estimation of N-Acetyl-D-Glucosamine (NAG) Content and Electrospray Ionization Mass Spectrometry (ESI-MS)

The Morgan–Elson assay was used in order to evaluate the *N*-acetyl-*D*-glucosamine released after chitinase treatment, as described previously. For more details see [6,11,12].

Sample preparation for the ESI-MS analysis was performed by the hydrolysis of organic matrixes obtained after HF-treatment of the biological samples in 6M HCl (24 h at 90 °C). The samples, after HCl hydrolysis were filtrated with a 0.4 micron filter and freeze-dried in order to remove any excess HCl. The standard D-glucosamine as a control was purchased from Sigma (Sigma-Aldrich, Taufkirchen, Germany Both the commercial standard and the prepared sample were dissolved in water before ESI-MS analysis. ESI-MS measurements were performed on an Agilent Technologies 6230 TOF LC/MS spectrometer (Applied Biosystems, Foster City, CA, USA) in line as a detector in the analytical HPLC instrument. Nitrogen was used as the nebulizing and desolation gas.

## 5. Conclusions

The results of this investigation showed the need to develop new, simultaneous, more effective methods of extraction of both biologically active compounds and chitinous scaffolds from *P. arabica* and other species. The possibility of farming of *Pseudoceratina* species from primmorph-based cultures and under marine ranching conditions possesses high potential for advanced blue biotechnology. Due to the fact that, the *P. arabica* species live at low depths (around 10 m) development of a new method for their aquaculture in tropical areas become very attractive from the industrial and economical point of view. It is already confirmed that chitinous scaffolds isolated from representatives of the order Verongiida are lucrative for the development of regenerative medicine. Further research could also be conducted to determine the possibility of technological application of chitinous scaffolds of *P. arabica* origin as advanced 3D composite materials under conditions of extreme biomimetics or adsorbents. We suggest that this study will trigger the future research dedicated to both (i) discovery of chitin within other representatives of the family Pseudoceratinidae (ii) and their utilization in modern technologies improving the quality of human life and health.
